# Heroes and Hysterics: ‘Partisan Hysteria’ and Communist State-building in Yugoslavia after 1945

**DOI:** 10.1093/shm/hku005

**Published:** 2014-05

**Authors:** Ana Antić

**Keywords:** war trauma, psychoanalysis, military psychiatry, Communist revolution

## Abstract

This article investigates a novel type of war neurosis defined by Yugoslav psychiatrists in the aftermath of the Second World War. This uniquely Yugoslav war trauma—‘partisan hysteria’—was diagnosed exclusively in Communist resistance soldiers—partisans—and did not manifest itself in the form of battle exhaustion or anxiety, as was the case in other armies. Rather, it demonstrated a heightened willingness to fight, and consisted of simulations of wartime battles. Yugoslav psychiatrists argued that ‘partisan hysteria’ most frequently affected uneducated and immature partisans, who were given important political responsibilities but experienced severe trauma due to their own inadequacy. I argue that ‘partisan hysteria’ served as an opportunity for upper-middle-class psychiatric professionals to criticise the increasing upward social mobility after the socialist revolution of 1945. Surprisingly, this touched upon an issue that had already provoked deep disquiet within the Communist Party, and resonated with the Party's own concerns regarding social mobility.

In 1945, the Yugoslav People's Army, having faced and triumphed over numerous formidable wartime challenges, was plagued by an internal problem which appeared impossible to resolve or even fully understand: a virtual epidemic of war neurosis, which affected thousands of partisan soldiers, and which did not show any signs of subsiding after the end of fighting. On the contrary, the end of the war seemed only to exacerbate the spread of the illness.^1^ This was a disorder that bore no resemblance to war traumas in the other nations that participated in the conflict: it did not manifest itself in the form of an urge to withdraw from the frontlines, as was the case in the British and US armies, where battle exhaustion, anxiety and demoralisation emerged as the most popular diagnoses by 1944. Rather, Yugoslav war neurotics demonstrated a heightened willingness to fight, as their new disorder consisted of violent and potentially harmful epileptiform seizures which simulated wartime battles and attacks. The seizures could occur at any moment and under any circumstances, usually when there was audience—in the middle of a conversation, at lectures or meetings, while driving or riding a car, in front of superiors, for example.

According to Dr Hugo Klajn, Vienna-educated Belgrade psychoanalyst and psychiatrist who treated a number of partisan patients in the immediate aftermath of the war, these involuntary seizures started when soldiers fell into a state of trance of sorts, during which they subjectively re-experienced intense feelings related to fighting. In addition, the partisans' seizures were generally preceded by distinct physical symptoms: ‘tightness in the chest, psychological confusion, weakness in hands, and unconscious closing of the eyes’. Klajn explained that, after experiencing varying initial physical symptoms,

the neurotic lays down … screaming: ‘Assault! Ahead, proletarians, brothers, fighters, comrades!’ or some similar combative outcry. His eyes are closed, breathing fast with loud expiration he raises his legs and hits the floor strongly, he hits himself in the chest, hits his head against the floor, raising fists. He imitates the position, moves and sounds of shooting from a rifle or some other weapon, throwing bombs. … Some, having calmed down a bit, give a speech to their comrades, reminding them of their sacrifices and achievements, complaining of those who have not treated them in a proper way.^2^

The seizures would end unexpectedly, and afterwards the patients generally did not remember what they had done or said.^3^ The illness rendered the partisan hysterics incapable of military service, disrupted their education or political activity, and could potentially result in severe physical injuries of spectators or the patients themselves. In addition, the partisan patients often openly disobeyed their superiors as well as the medical personnel, thereby arousing fears that the epidemics of partisan hysteria would result in mass indiscipline and social chaos. But partisan resistance fighters were the foremost military and political heroes of socialist Yugoslavia, and both the psychiatric community and the political authorities were aware that the discussion regarding the symptoms and their implications had to be conducted with utmost care.

Even more importantly, only certain ranks of partisan soldiers seemed to be affected by the illness. By 1945, the partisan neurotic appeared to be a precisely defined type with a very distinct (low) socioeconomic position, the serious psychiatric repercussions of which seemed to clearly demonstrate the dark side and subversive potential of increased social mobility. The Yugoslav form of war neurosis apparently most frequently affected the uneducated, socially immature and emotionally less sophisticated—in some reports even ‘primitive’—members of the partisan troops, who were given important political responsibilities but experienced severe trauma and anxiety due to their own inadequacy and unpreparedness. Klajn's case files, for instance, all began with the patient's age and educational level, always mentioning illiteracy if it was present, in order to support his claim that partisan neurosis was virtually only diagnosed in extremely young, uneducated (frequently illiterate) and immature soldiers, whose limited intellectual capacities frequently clashed with highly responsible assignments that they had been given (or to which they aspired) towards the end of the war. This in turn created internal conflicts and feelings of guilt and dissatisfaction.

In this article, I will explore the post-war psychiatric and socio-political discussions of the diagnosis of ‘partisan hysteria’, and illustrate how they reflected the political exigencies and dilemmas of the newly socialist Yugoslav society. I trace why this new category emerged and how it was related to the complex socioeconomic and political circumstances in Yugoslavia and East Central Europe after 1945. I argue that ‘partisan hysteria’ served as an opportunity for middle-class or upper-middle-class psychiatric professionals to express their anxiety over, and even open disapproval of, the increasing upward social mobility and related socio-political transformations following the socialist revolution of 1945, and to criticise the effects of the post-war creation of a new political and military elite from the ranks of workers and peasants. Given the greater visibility and social authority of the urban and rural poor from 1945 on, ‘partisan hysteria’ gave psychiatrists an opportunity to define this new source of social instability, and devise ways to solve it—through education, control or limitations of upward social mobility. In other words, witnessing a true social revolution outside the hospitals, Yugoslav psychiatrists found it very difficult to shed their long-time beliefs about the volatile nature of the ‘masses’, and pathologised the very upward mobility and other revolutionary societal changes of the new Yugoslav socialist republic. This was true not only of the more politically conservative members of the profession, but also of some of the most left-wing and progressive psychiatrists and psychoanalysts.

At the same time, ‘partisan hysteria’ touched upon an issue that had already provoked deep disquiet within the Communist Party itself. After 1945, the Party leadership was concerned that the lax wartime entrance criteria to its ranks had resulted in a large influx of members of questionable political and ideological ‘value’, which compromised the Party's ideological character. The new rulers were increasingly worried that the recent recruits might not be well prepared for their tasks in the post-war reconstruction. The psychiatric discussions thus became both politically subversive and extremely important for understanding the problems with which the Party itself was dealing. The psychiatric research on war neurosis, while potentially very dangerous to the reputation of the victorious partisan army, also interrogated and offered solutions to the problems that the Party ideologues themselves considered to be of utmost significance—namely, the idea of Marxist revolution in the heavily agricultural and underdeveloped Yugoslavia presented a number of problems of various kinds, and posed serious ideological dilemmas to old Party members who worried about the Party's ability to implement radical socioeconomic and political measures.

My article contributes to the growing historical scholarship on war psychiatry. While there exists vast academic literature on shell-shock in the first Wolrd War, the impact of the second World War on psychiatric reformulations of the concept of war trauma remains under-researched, while still fewer historical studies have appeared about the Second World War psychiatric casualties.^4^ It was not, however, until the unprecedented psychological effects of the Second World War on both civilians and soldiers that more radical and long-lasting transformations occurred within the dominant organic psychiatric paradigm. Moreover, it was only after the devastating experiences of the Second World War that some of the core psychiatric lessons of the First World War were finally incorporated in the discipline's theoretical and therapeutic framework.^5^ My article addresses this gap in scholarship. I examine how the experiences of 1941–5 transformed psychiatric perceptions of the human psyche and re-shaped the profession's understanding of the origins and therapy of war neurosis in Yugoslavia, a country which, by 1945, had survived one of the most brutal Nazi occupation systems in Europe as well as a fully successful socialist revolution. At the same time, this is the first study of the history of Yugoslav (and Balkan) Second World War psychiatry and war trauma.

This research also addresses the fraught relationship between psychiatry and political revolution. As Paul Lerner's book demonstrates, psychiatrists in Central Europe tended to align themselves with conservative political forces, and diagnosed appeals for radical socioeconomic and political transformations as symptoms of mental disorders, while also enumerating negative psychological effects of revolutionary systems such as the Weimar Republic.^6^ Pre-revolutionary Russian psychiatry, on the other hand, argued that autocracy stunted psychological development and produced pathological reactions in the population.^7^ While many valuable studies have analysed psychiatrists' orientation in favour of or against revolutionary politics and changes, my article explores a situation in which (progressive) psychiatrists and the revolutionary Communist Party itself shared deep concerns regarding the revolution's pathological potential. It reveals the complexity of psychiatric evaluations of the political turmoil of 1945, and shows how psychiatry, short of denouncing the revolution entirely as detrimental to mental stability, could express the Party's own worries about the chaos which the revolutionary change might encourage. In this sense, my research offers a different view of the relationship between psychiatrists and revolutionary politics, one in which there were no clear alignments or expressions of uncompromising opposition or support. By criticising dangers of the revolutionary chaos and the population's unpreparedness for taking over new responsibilities, psychiatrists recommended themselves as the optimal actors to resolve the tension, adjusting their pre-revolutionary ‘enlightenment’ mission to the new circumstances. At the same time, a history of ‘partisan hysteria’ and its complex and contradictory interpretations reveals a significant aspect of the social and political context of post-war Yugoslavia, and indicates hitherto unexplored avenues of research into the Communist Party's own misgivings regarding the revolutionary changes.

## Historical Background and Politics of Psychiatric Treatment

In many ways, partisan hysteria turned out to be a disorder which threatened not only military efficiency and national security, but also the very foundations of the new socialist society. It both compromised the soundness and reliability of the victorious partisan soldiers, and made it difficult for the young state and the Army to complete the transition to peacetime conditions—in particular to realise the aim of overturning the existing political elites and advancing new ones from the ranks of peasants and workers. In addition, ‘partisan hysteria’ was overall much more violent and therefore disruptive than any form of battle exhaustion or hysterical shivering, and the ill partisans were increasingly seen to be dangerous to public order.

The disruptive military and political features of partisan neurosis are illustrated in Klajn's post-war case notes. In 1945, for instance, Klajn treated Mile M., a 27-year-old illiterate peasant, who had joined the movement in early 1942 after having survived the murder of his entire family and spending time in a concentration camp, and who started having hysterical seizures in 1944. Mile emphasised that all the doctors who treated him ‘said that he could only be cured if he had his “every wish fulfilled”’. Klajn also noted that the patient lacked discipline, refused any work ‘as he considers himself ill. To any request or objection he reacts with a seizure.’ At the end of one seizure, Mile delivered a speech, in which he stated that ‘I didn't get this [nervous seizures] in someone else's barn but in the fighting. (Beats his chest). Dear comrades, my beloved soldiers, honour to our dead and disabled fighters, and to those suffering neurosis. We shouldn't work.’^8^

Klajn emphasised the difficulties that the patient had adapting to the post-war circumstances, in which he was required to adopt new skills and invest greater efforts. Prospects were rather bleak, however, as his illiteracy inevitably became an enormous obstacle to his further advancement—something that only became evident after the fighting was over. In Klajn's interpretation, patients like Mile felt embittered as they expected rewards for their wartime achievements and sufferings instead of having to deal with a number of peacetime tasks which they simply could not fulfil due to their limited capacities. The trauma of the transfer from ‘his unit, in which everyone knew and appreciated him, to … an environment in which he simply had to feel unacknowledged and marginalised due to his lack of education’ was overwhelming.^9^ Klajn's description of the case of another 20 year old illiterate peasant, Niko N., demonstrated this even more clearly. As Klajn reported, Niko stated that he first started getting seizures because he was ‘“unnerved that the war had ended”, because now soldiers were facing tasks which he, as an illiterate person, could not and would not fulfil. He wished the war was still going on.’^10^ Salko B., also 20 years old and illiterate, was particularly bitter because, at political classes organised by the Party in order to raise the educational level and ideological awareness of its members, he was not able to take notes like the others. Like Niko and Mile, Salko seemed to have seizures rather frequently, usually whenever faced with an unsatisfactory situation or negative criticism. ‘Partisan hysteria’—described in this way—thus exposed some major flaws within the military, and also brought into question the viability of the new political and ideological tenets.

Klajn found the soldiers' unpreparedness for peacetime socialism deeply upsetting, and his interpretations were also highly critical of the Army's attempts to handle the problem. His approach to his partisan patients and their psyche remained respectful, and he never questioned the value of their wartime contributions. However, although Klajn was the core practitioner engaged in the treatment and theoretical discussion of partisan hysteria, he was not the only one: the issue eventually involved a network of military and civilian psychiatrists and psychoanalysts. Unsurprisingly, a complex and wide-ranging psychiatric debate ensued regarding the origins of the neurosis, and the psychological character and motivations of the neurotics, whose behaviour thoroughly upset the Yugoslav military's discipline and internal organisation. In these debates, two camps of psychiatrists emerged, whose interpretations and proposed therapies differed radically. On the one side, a group gathered around Zagreb's leading military psychiatrist Lieutenant-Colonel Josip Dojč, approached the partisan patients as thinly veiled deserters and shirkers, feeble-minded or psychopathic personalities, comparable to spoiled and ‘unreeducable’ children, and discussed the disorder exclusively in terms of oligophrenia and simulated hysteria. On the other, a circle of psychotherapeutically oriented civilian psychiatrists, most importantly Klajn and his colleague from Zagreb Stjepan Betlheim (who subsequently changed sides), defined the diagnosis in more positive and sympathetic terms, without belittling their patients' dedication.

The roots of this psychiatric disagreement regarding the nature and motivations of the partisan hysterics lay in the medical experiences of the war time years. The Yugoslav Army's medical personnel first noted this epileptiform disorder in autumn 1943, and initially termed it ‘Kozara hysteria’ [*kozarska histerija*], after the mountain area where the seizures first occurred. By the spring of 1942 the partisan movement had achieved significant strength and liberated sizeable territories, but in the second half of 1942 and in 1943 it suffered some of its worst losses in a succession of the Wehrmacht's pacification and cleansing operations in Bosnia, Montenegro and Croatia. The battle of the Kozara mountain, in northwestern Bosnia, became the symbol of partisan suffering and dedication, a place where the partisans were heavily outnumbered, encircled and brutally executed or taken to concentration camps together with tens of thousands of local civilians. The autumn of 1943, however, became the turning point, and after the capitulation of Italy the partisan army gradually recovered its positions and ultimately emerged victorious. Remarkably, it was only after the worst defeats and dangers were behind, and the partisans could be safely evacuated to free territories, that ‘partisan hysteria’ first appeared, and this would figure significantly in subsequent medical discussions. Until well after the end of the war, the Yugoslav authorities had not agreed upon a preferred therapy for the partisan patients, and had not decided whether the neurotics should be removed from their units in the first instance.^11^ The initial response was to take the illness seriously, and send the affected partisans to hospitals on the Italian territory or in the liberated areas in Yugoslavia. However, as the number of diagnosed neurotics rose—ultimately affecting around 5,000 soldiers—many military physicians and psychiatrists soon suggested that the best approach would be not to ‘pamper’ the partisans, and not to send them too far from their units, and by 1946–7 this opinion prevailed in military psychiatric circles.^12^

Military psychiatrist Isak Alfandari, who worked with dozens of partisan mental patients recovering in Italy during the war, claimed that the core motivator for this type of soldiers' neurosis was the wish to remove oneself from the battlefields.^13^ Dojč went even further: he sarcastically termed the disorder *Inozemstvo-mania* [foreign lands mania], which implied that the soldiers merely wanted to reach the comfort of the free Italian territory—and applied rather harsh therapeutic methods, such as electric shock treatment, pouring cold or hot water over patients during seizures, administering strong anaesthetics, etc.^14^ On the other hand, Dr Šalek, head of the mobile hospital of the Fourth Division of the Yugoslav Army stationed in the area where the disorder was first reported, wrote from the frontlines that the prognosis for a full recovery was exceptionally good, and this in itself was a proof (and consequence) of the soldiers' bravery and self-sacrifice: ‘all or at least a majority of the neurotics gradually … start to re-experience a need for a community to which they had been so tightly related ideologically during the war. In their minds, altruism again overpowers the selfish instinct.’ Šalek added that it was precisely the courageous, experienced, most dedicated soldiers who most often suffered from ‘partisan hysteria’, and that the wish to escape could not have been further from their minds.^15^

This difference of interpretation between Betlheim and Klajn on the one hand, and the traditional military psychiatrists on the other, signified a deeper and long-standing rift in Yugoslav psychiatry between those practitioners dedicated to the biomedical model and the psychodynamically or psychoanalytically oriented ones.^16^ Quite tellingly, both Klajn and Betlheim indicated that the source of the partisans' illness was in an unconscious conflict—between the soldiers' honest commitment to their military (or ideological) duty and their subconscious wish to save their lives or accrue rewards, that is between selflessness and more self-centred motivations. According to Betlheim, ‘there is no doubt that there are in him [partisan neurotic] unconscious strivings for safety, for protecting his own ego, but those wishes cannot be experienced consciously, because his entire morality—a result of his upbringing, and especially of the earlier military camaraderie—would militate against such tendencies.’^17^ By contrast, military psychiatry wavered between viewing the neurotics as constitutionally flawed—weak-minded recruits who should have been weeded out in the first place—or treating them as simulants and liars, who should be punished instead of rewarded by being taken seriously. The military psychiatric interpretations approached the issue of partisan neurosis in static terms, offered very little in the way of therapy, and refused to pay any attention to the ill soldiers' psychological conflicts or to their wartime experience of most severe psychological traumas.

In the context of these psychiatric debates, psychoanalysis emerged as the dominant framework to interpret and treat partisan neurosis. It was very striking that, in the immediate aftermath of the war, the therapy and theory of partisan neurosis were officially entrusted to Klajn and Betlheim. In 1945, Klajn and Betlheim were placed in charge of the Military Psycho-hygienic Institute in Kovin, a former mental hospital in the vicinity of Belgrade, which was supposed to develop into a specialised mental health centre for the treatment of the partisan neurotics (this therapeutic experiment was discontinued after only a few months due to limited resources as well as the apparent inability of the Institute's staff to maintain order and discipline).^18^ Partisan neurosis thereby became the professional domain of psychoanalysts, a diagnosis upon which Yugoslav post-war psychoanalysis would attempt to establish itself as a relevant discipline, while this was the very first time that Yugoslavia's military psychiatry officially instituted any form of psychotherapy or resorted to a psychodynamic model of mental illness.^19^

Psychoanalysis—thoroughly marginalised and often ridiculed in interwar Yugoslavia—first entered the official psychiatric circles through the figure of the partisan hysteric, and it was a grand entrance. In an important sense, it was understandable that psychoanalysis succeeded in securing such a central place for itself in the therapy and professional discussions of partisan neurosis: both Klajn and Betlheim initially approached the problem with infinitely more understanding for their patients than traditional psychiatrists did, and, even more importantly, with much greater therapeutic optimism. Writing these patients off would have had enormous political ramifications. Since psychoanalysis, or at least a psychoanalytically informed psychiatric approach, did not explain partisan neurosis in terms of flawed heredity and untreatable constitutional predilections of such a large number of members of the victorious Yugoslav liberation military, it gave its practitioners a significant advantage in the new socialist republic. Moreover, as will be explained in some detail below, Klajn and his followers argued that the extraordinary, unmatched character of partisans' war neurosis reflected some unique and positive traits of Yugoslavia's socialist military (and society), and this emphasis on Yugoslav exceptionalism made the psychoanalytic approach politically viable, even in the face of decisive opposition from the military psychiatric circles.

This newly acquired respectability of psychoanalytic practitioners reflected the ongoing change of paradigm in the Yugoslav psychiatric circles. In interwar Yugoslavia, university and hospital psychiatry had been firmly embedded in the Central European biomedical framework, and virtually no psychotherapy or psychoanalysis was practised and taught outside individual private practices.^20^ As head of the Zagreb psychiatric hospital Dezider Julius wrote in the first years after the war, psychoanalysis provided essential therapeutic insights without which pre-war, bourgeois, organicist psychiatry could not be properly reformed in a new socialist setting, primarily because it required the practitioners to take into account environmental, that is socioeconomic, factors in the onset of mental illness.^21^ Still, the precarious position of psychoanalysis in socialist Yugoslavia almost certainly plagued Klajn's and Betlheim's professional engagement with the partisan neurotics. From a Marxist point of view, the ideological shortcomings of Freud's postulates, his problematic ‘idealistic-philosophical’ deviations, remained an enormous obstacle to a proper integration of psychoanalysis in the official psychiatric institutions. Thus, in spite of the initially privileged position of both Klajn and Betlheim, there were many reasons why the psychoanalytic conceptualisations of this unique war trauma were a highly sensitive topic.^22^

## ‘Yugoslav partisan neurosis’: Exceptionalism and its Discontents

As we saw, the dilemma whether ‘partisan hysteria’ indicated cowardice (and mental pathology) or a deeply heroic commitment had already been formulated well before the end of the war, and it immediately became tightly intertwined with the issue of the disorder's supposed national and geographical uniqueness. Initially, the distinctiveness of the epidemic seizures of the partisans was denied, and attempts to discuss the disorder in ethno-psychiatric terms were criticised.^23^ On the other hand, already in 1945 a group of psychoanalytically oriented psychiatrists, led by Klajn and Betlheim, started offering interpretations and descriptions of the disorder that heavily relied on notions of Yugoslav exceptionalism, and directly related the diagnosis to both the Yugoslav ‘national character’ and the egalitarian democratic nature of the new people's army. These doctors constructed the diagnosis of ‘partisan neurosis’ or ‘partisan hysteria’ (used interchangeably) as expressive of certain social and cultural traits specific for (socialist) Yugoslavia. The change of rhetoric occurred, therefore, soon after the war, when the number of partisan neurotics continued increasing at an undiminished rate. While those military psychiatrists who treated the partisan neurotics at the early stage—that is, during the war—gradually grew to believe that the problem was merely temporary, and that, moreover, too much medical attention would not help the neurotics to overcome their state, the post-war situation indicated that the disorder was there to stay.^24^

In some of these later accounts the notion of partisan neurosis acquired positive connotations: it was arguably a disorder of dedicated fighters, who considered their own interests to be identical to those of their army or of the new socialist republic. Partisan neurosis or partisan hysteria could thus become an advertisement for the new kind of society and people's democracy that the Yugoslav authorities were building. Marxist politics eliminated trembling, anxiety and battle exhaustion, and created soldiers who, even when faced with the worst traumas and suffering from shell-shock, kept their devotion to the army's cause, and their willingness to remain on the frontlines.^25^ Betlheim argued that it would be an offence to consider the neurotic ‘a person who consciously strives to escape the war efforts and who consciously wants to withdraw to the rear’, because the majority of the patients ‘had been through difficult experiences which they had barely survived. … They often, having invested the last vestiges of their strength, saved themselves from the dangerous situation, and then had their first seizure in a safe territory.’^26^ In this psycho-dynamic discourse, the local specificity of the disorder resulted from the superiority of the Yugoslav resistance movement in comparison with both the Allied and the enemy armies in the Second World War, and the conditions of Yugoslav soldiers in the First World War.

In Klajn's writings, this was posed as the central question for the analysis not only of partisan hysteria but of war neurosis in general: what accounted for the difference between the Yugoslav and the Allied soldier neurotics' reactions, and what could explain the former's continued dedication as opposed to the latter's loss of motivation and battle exhaustion? For Klajn, the peculiar ‘fighting spirit’ of the Yugoslav partisans naturally produced an illness which centred around demonstrations of aggression and attack, instead of more direct expressions of fear such as trembling, anxiety, withdrawal, and paralysis. In an army made up of volunteers—people who virtually had to take up arms for survival—the urge to withdraw from the battlefield, the feeling of anxiety about continuing in the war (which played a crucial role in the development of the ‘anxiety state’ or ‘battle fatigue’ in the US and British armies) necessarily held much less significance. In the case of the two Western armies, ‘the main pathogenetic and pathoplastic factors were fear and desire to withdraw from the area of war danger’, while the most widespread type of the Yugoslavs' war trauma ‘was not a neurosis of tremor and fear, but a neurosis of combat, of storming’.^27^ In this way, Klajn's definition preserved the image of resistance fighters as courageous, self-sacrificing soldiers, fearless in the face of superhuman hardships—a myth of national proportions that was being constituted as he wrote:

[s]ince they did not join the war as a result of an order or against their will, they don't react with fear to the wartime horrors; they were led [to the battlefields] by an irresistible urge to murder the murderers of their families … they can react even to the worst with increased pugnaciousness.^28^

Moreover, the working-class, anti-capitalist ideological character of the Yugoslav resistance further determined the nature of psychological reactions: our soldiers, he wrote, ‘gathered to realise a clearly set goal, unified by a common interest; the interest of a capitalist state, on the other side, differed from the interests of the majority of soldiers—to the contrary, those who were the most interested in the war and its result were mainly far from the battlefields—and that is why the American soldier “fought because he had to”’; hence his ‘negative disposition, indifference and “insufficient motivation”’.^29^

However, it soon became clear that the argument in support of uniqueness could have its negative sides as well. In the years after 1945, many of the socio-political and cultural problems appeared that would mould the interpretation of the diagnosis, and make it a useful site for broader social criticism. Many psychiatrists who did not share the sympathetic attitude (such as Josip Dojč) could and did still subscribe to the definition of partisan neurosis as uniquely Yugoslav, but their discussions of what was now considered a typical partisan disorder expressed their harsh criticism of the new Yugoslav realities, the partisan guerrilla army and its troubled integration into the new state. On the other hand, even those psychiatrists who showed most respect towards their partisan neurotic patients tended to view the disorder as an example of some of the most serious problems which beset the post-revolutionary society in Yugoslavia.

As I will show below, Yugoslav psychiatrists described and evaluated the partisan neurotic in a number of often contradictory ways. At different times, and in different doctors' articles, he or she was viewed as: a brave, self-sacrifising fighter; a misbehaving child with a poor upbringing; an aggressive and rebellious troublemaker; or a weak-minded recruit who should have been weeded out in the first place. But quite surprisingly, all these conflicting interpretations had in common a certain image of a typical partisan neurotic and his core attributes: a person strongly characterised by the lack of education (often of literacy) and poor social background; violent reactions to negative criticism; over-ambitiousness; inability to adjust to peacetime conditions; insubordination; immaturity; and ‘infantilism’. Both psychoanalysts and traditional military psychiatrists often discussed the partisan neurotic in terms of ‘primitivism’ and a lack of cultural, political or social sophistication. Whether they viewed the partisan neurotics as valuable and ‘curable’ soldiers, or as unfit recruits with psychopathological characters and limited intelligence, virtually all psychiatrists dealing with this problem, whether from an organicist or a psychodynamic perspective, agreed that these partisans' psychological problems demonstrated the core social and cultural difficulties of Yugoslavia's post-war revolutionary transition. Since they were often considered to be the socialist republic's military and political elite in the making, their mental breakdowns attracted attention because they could be seen to point to the inadequacies and dangerous potentials of the new order. The constructed psychological profile of the partisan neurotic thus exemplified a number of troubling socio-political tendencies of the immediate post-war era, and psychiatric discussions of this diagnosis united criticisms of several broader societal trends: radically increased social mobility and the new elite' unpreparedness (incompetence) to take over the new duties; violent potentials of such revolutionary elite turnover (as showed in psychiatrists' concerns over the neurotics' insubordination to psychiatric authority); revolution in sexual mores; and radical changes in the position of women, both in the partisan guerrilla army and in the Yugoslav society.

## Social Mobility: Enlightenment or Exclusion?

Klajn made sure to define the neurosis in such a way as to not compromise his patients' dedication to military struggle, but he still concluded that the illness was a ‘sign of a certain slowdown in development, certain infantilism’, or an ‘underdevelopment of character’.^30^ But for him, the problem was not the incapacity itself. The original conflict was actually determined by the patients' desire to fulfil their new tasks, and by their exceptional ambition for professional and political advancement and recognition. In this respect, Klajn's work addressed the issue of greatly increased social mobility in Yugoslavia after 1945 in a very complex way, and delineated its broader social and psychological consequences in a less than positive light. The unique nature of the partisan army organisation was that it offered an unprecedented opportunity for people from the lowest sectors to achieve high-ranking, responsible and socially prestigious positions. Their capacity to succeed in their new tasks was doubted by virtually everyone: the Party, psychiatrists and, finally, themselves. For Klajn, this was one of the main sources of neurotic reactions: this ‘need to make independent decisions in a number of tasks, and thereby take personal responsibility for their solutions’ had a particularly strong ‘pathogenic effect’ on those soldiers with ‘immature characters’, who were ‘perhaps also intellectually and otherwise less than developed’.^31^ In addition, the very possibility of achieving professional and social success stimulated in many partisan soldiers extraordinary ambition and a very powerful desire to be rewarded. When peacetime circumstances made the achievement of that recognition more difficult or even impossible, soldiers resorted to hysterical seizures as (immature) forms of protest, or as a roundabout strategy for realising their goals.

In fact, Klajn highlighted the ‘wish for being recognised’ as the single most important psychological factor in the development of partisan neurosis: this also explained why so many new cases were registered after 1944 and 1945. While this wish could easily be satisfied during the war in battles (through self-sacrifice and consequent admiration by comrades, commanders and the local population), the circumstances after the end of the war offered fewer opportunities for immediate acquisition of rewards and praise. Consequently, ‘neurosis represented a promissory note for that type of recognition, seizures—a dramatic display of one's claims, of one's (under-appreciated and unrewarded) achievements and sacrifices, much more effective than mere talking about them would have been.’^32^ Moreover, this was the reason why virtually no partisan neuroses had been recorded before 1943. The distribution of officer ranks, distinctions, and status rewards within the victorious army in the spring of 1943 was held responsible for the seizure of many ‘incompetent’ and overly ambitious partisans, who found themselves in lowly positions within the hierarchy: these changes ‘incited envy and awoke ambition and desire for rewards among the partisans, especially in uneducated, young and psychologically immature soldiers’. When advancement was denied or jeopardised, ‘the wish emerged in immature and vain partisans to vent their anger and receive what they thought was a deserved award’.^33^

Therefore, in the final analysis, partisan neurosis was *the* typical mental condition of a highly socially mobile community: the neurosis was the Yugoslav socialist society's ‘infantile disease’. This idea was perhaps expressed most clearly in Klajn's description of the case of Misa M., a 20-year-old non-commissioned officer, who in 1945 started suffering seizures while attending a radio-telegraphic course in which his results were unsatisfactory, and also had one ‘at a political class when a comrade criticised his statements. He is very ambitious, wants to remain a political official’.^34^ In that sense, Klajn's work criticised the wartime radical politics and social mobility of the partisan units, which were being translated into a post-war social system.

The expectation that this created in unstable and immature persons frequently drove them to aggression and indiscipline. This became particularly clear to Klajn and Betlheim while they were involved in a failed attempt to treat around a hundred partisan war neurotics at the Military Psycho-hygienic Institute in Kovin. In his descriptions of his experiences in Kovin, Klajn indicated the potential social danger of such a strong ambition awoken in the lower classes. In Klajn's account of some patients' behaviour, the anticipation of an imminent eruption was clearly present: Niko N. ‘is permanently dissatisfied, walks around with a stick, threatens and stirs up others … he leaves the Institute on his own, does not recognise the commissar as his superior’;^35^ Jovan O. is ‘undisciplined, leaves without permission and returns late … he broke a window. … Threatens the superintendent and the clerks.’^36^ Klajn also explained how the ‘fighting spirit’ that, according to his interpretation, characterised this particular neurosis, made the patients ‘very unpleasant’, inclined to act violently, attack the medical and administrative staff at the facility where they were placed for treatment, behave extremely disobediently, participate in beatings, and break and destroy. Klajn reported that five particularly undisciplined soldiers even threatened to murder all members of the Institute's management. He remained resolute in his claim that the issue of partisan neurosis was a social problem much larger than ‘neuropsychiatry itself, and which also falls within the scope of social psychology and politics, pedagogy, military discipline, military court system, even criminology.’^37^ In other words, the source of the neurosis was to be found in some of the most widespread social circumstances, and the challenge that the disorder presented was certainly not only medical, but affected a number of other aspects of the new society. Klajn's and Betlheim's disconcerting experiences in the microcosm of the Military Psycho-hygienic Institute demonstrated partisan hysterics' potential to permanently upset social order and to develop into an uncontrollable factor in a larger social setting.^38^ This image of destruction and chaos that resulted from the fear of the lower classes taking over thus persisted after 1945: the social revolution had its apocalyptic potential.

Klajn's solution was ‘pedagogical therapy’, the aim of which was to re-educate the core of the patient's personality: the correct attitude of the broader society to the patients should be that ‘of a mature educator towards an immature pupil’.^39^ The upwardly mobile illiterate peasant or unqualified worker was constructed as an unruly student, whose behaviour needed to be put in order by a sympathetic yet firm and authoritative teacher-psychiatrist. Therefore, if the newly emerging social elite was lacking a proper upbringing and education and needed to be enlightened, the psychiatrists reserved that role for themselves from the outset. Klajn made it clear that in his opinion it was ‘likely that the unenlightened nature of our peoples contributed to the spread of hysterical reactions in this war’.^40^ The enlightenment, therefore, was the best prophylactic measure. Klajn emphasised not only the need to educate the broadest social sectors about mental health and genesis of psychiatric illness, but also to engage in a much more all-encompassing agenda for popular edification.

As we saw, a group of military psychiatrists based in Zagreb chose a significantly different set of terms to define the partisans' war neurosis. In their case, the professional goal of strengthening the position of psychiatry and psychiatrists within the newly emerging military establishment determined their medical and therapeutic involvement with this issue, so that, in their arguments and recommendations, education gave way to exclusion. In other words, they called for a much stricter selection of military recruits and functionaries, rather than for their enlightenment and improved upbringing, primarily because the processes of selecting the valuable and excluding the ‘challenged’ from military service would necessarily have to be conducted by military psychiatrists, whose numbers and influence would consequently grow. In this sense, as civilian psychiatrists tried to recommend themselves as primary educators of the newly empowered masses, their colleagues from the military became invested in raising the entrance criteria and promoted their own central role in it.

Dojč certainly found Klajn's image of the neurotic as a misbehaving child very pertinent, although his solutions and the implications of his work were considerably more radical. In Dojč's opinion, the most common war neurotic was a ‘young infantile and primitive’ person; their neurotic seizures could be compared to ‘some sort of infantile reaction of spite, similar to those by ill-bred small children, if parents don't fulfill their wishes. In the same way, these children throw themselves on the floor, cry, scream, hit around with their hands and legs, in order to provoke pity, compassion or concern of those around them and achieve their goals in that way.’^41^ Even Betlheim, who later earned fame as one of the most prominent proponents of the psychodynamic treatment of neuroses in Yugoslavia, at this time developed a close professional relationship with Dojč and a group of military psychiatrists in Zagreb, and seemed to have adopted his colleague's harsher, more pessimistic stance following the disappointing therapeutic experiment in Kovin: ‘it is known that the psyche of a hysteric is in many ways similar to the psyche of the underdeveloped, that there are many infantile traits in it. Our war neurotics are mostly youngsters, people with unfinished puberty … similar to spiteful children, who throw themselves down, bite themselves and others, pull themselves and others for the hair etc.’ Furthermore, they were ‘full of theatricality in a primitive way’, and their urges were ‘egocentric’ and ‘autistic’.^42^ The change in rhetoric was slight but noticeable: Dojč's and Betlheim's terms were more directly derogatory and dismissive of the legitimacy and complexity of the patients' internal conflicts.^43^

While Klajn emphasised the need for development and edification, Dojč and his associates defined their partisan patients in static terms—as frequently pathological personalities, whose behaviour during seizures differed very little if at all from their conscious selves.^44^ In his own practice in the Zagreb Psychiatric Military Hospital, Dojč remained firmly within the biomedical framework of interwar Yugoslav psychiatry: war neurotics were often also diagnosed with psychopathy (or *psychopathia gravis*), and their intelligence was reportedly measured in the lower eighties (according to the Binet-Simon test). In his notes and diagnostic explanations, he took no account of the partisan neurotics' often extreme psychological traumas (many had been severely wounded multiple times, participated in incessant fighting against a much stronger military force for over two years, and survived bombings, raids and concentration camps). Instead, Dojč focused on constitutional flaws, family history of psychiatric disorders and feeble-mindedness and organic predilections for psychopathic or hysterical reactions.^45^ At a talk given before a group of senior military psychiatrists in Zagreb in 1946, Betlheim even adopted an overtly eugenic stance: he argued that partisan war neurotics were ‘persons who generally exhibited irregularities of character’, frequently used seizures to express their aggressive or criminal tendencies and sadomasochistic complexes, and ‘usually were not suitable breeders of future generations’.^46^

Consequently, military psychiatry offered no psychotherapy at all. In a long article on the simulation of psychological disorders, Dojč concluded that the experience with the partisan neurotics proved their ‘psychopathic disposition’, which could not be cured. The only viable therapeutic option, he continued, was for the psychopaths from the partisan ranks to be ‘forced’ to behave ‘socially’. In order to eliminate the epidemic of the disorder, the psychiatrists needed to assume a ‘firm pedagogical stance’, instead of treating their patients as truly ill, because ‘psychopathic or hysterical reactions tend to express themselves in the form of simulations’.^47^ For these reasons, the suggested therapy did not include enlightenment; Dojč insisted on harsh disciplinary measures—punishments dispensed not only by psychiatrists but also by the political and military authorities, for the patients to internalise the accepted norms of behaviour, and to ‘learn very quickly that in this way they cannot reach their goal’.^48^ In order to encourage their ‘will to suppress their psychological weaknesses and asocial tendencies’, a proper punishment was necessary to make the patients ‘try to behave in a disciplined way … not to succumb to their weaknesses and become recidivist’.^49^ Colonel Dr Lavoslav Glesinger argued that the ‘difficult problem’ of the epidemic of wartime hysteria was partially solved when ‘the order came that war neurotics were not to be considered ill’. Like Dojč, Glesinger heavily relied on various techniques to interrupt the seizures and persuade his patients, more or less forcefully, that theirs was not a legitimate medical problem.^50^

As a military psychiatrist, Dojč told a different story about the wartime partisans than Klajn, as he was particularly concerned with the problem of mentally unfit soldiers accepted into the army. As a result of wartime necessities, in his view it was likely that a significant number of people whose mental abilities precluded satisfactory service and advancement in the army had nevertheless been admitted and even promoted within the partisan ranks. Problems emerged when, after autumn 1943, the partisan army saw a quick spread of the seizures related to war trauma, which turned into a true epidemic and raised the issue of the overall quality and fitness of partisan soldiers. Dojč saw this pull of people—‘psychopaths’, ‘neuropaths’, ‘hysterics’ as well as the ‘weak-minded’ or the ‘intellectually insufficient’—as the most likely to be diagnosed with hysterical seizures of the partisan type. The discussion of war neurosis in the partisan units thus offered an excellent opportunity to emphasise that the discipline of psychiatry was vital for the smooth functioning of a modern military in any future war, which was bound to be fought with weapons so destructive and techniques so psychologically shattering that neurotic and psychotic breakdowns would present the greatest obstacle to victory. Dojč tried to demonstrate the complications that the absence of psychiatric screening of recruits could create, and his discussion and treatment of the partisans' war trauma were significantly shaped by this professional concern. In consequence, he focused on the constitutional inability of potential and actual soldiers and how to eliminate the mentally ‘insufficient’, and in the process paid very little attention to the developmental potentials of his patients: he was clearly not interested in the issue of raising the educational and cultural level of the newly emerging military elite.

In his work, Dojč emphasised the need to avoid assigning great responsibilities to people of limited intellectual capabilities and insufficient cultural and educational preparation. In opposition to Klajn as well as the Communist Party itself, Dojč urged the military and political authorities to recognise the intellectual limits of the rural and urban poor. The issue of partisan neurosis functioned very well to show what happened when responsibilities and socially prestigious positions—or their prospects—were heaped upon the intellectually unfit. The existence of such great numbers of mentally challenged and psychopathological personalities in the current ranks of the Yugoslav army dictated the urgent need for a more extensive military psychiatric service capable of recognising the problematic types and assigning them their proper roles—inside or outside the army. This was necessary in order to identify those who were psychologically and intellectually ‘deficient’, and ‘interrupt their useless military education in a timely fashion and save the unnecessary costs of their schooling’.^51^ Edification, therefore, was not always a proper institutional response: in some cases, instead of becoming high-ranking military officers, the ‘intellectually backward’ and weak-minded could have been much more useful for society in ‘peaceful practical occupations’ such as ‘agriculture, raising livestock’, or some other form of simple physical labour.^52^ Education was certainly a waste of resources not only in the case of those genetically intellectually challenged, but also with regard to those persons whose intellectual development had been stalled because they had had no access to educational opportunities. For Dojč, it was necessary to realise the ‘natural’ limitations of upward social mobility.

In order to preclude the recurrence of such problems in the future, it was necessary to increase the educational level and numbers of military psychiatrists, who were the sole authority capable of deciding which potential soldiers would be fit for military service. Since, according to Dojč, in the Second World War psychiatric disorders accounted for the largest number of dismissed or incapacitated soldiers, he opined that psychiatry consequently became the foremost discipline in military medicine, as significant as war surgery or internal medicine: ‘we may not forget that we are, as they tell us, in a “cold war,” and a cold war means a “war of nerves.” … For these reasons, the one who wants to win the war must have not only better technology but also better nerves.’^53^

## The Communist Party and the New Man

The psychiatrists' concern with radical changes in upward social mobility after 1945, and their negative assessment of the capabilities of the new Yugoslav political and military elite, did not fall on deaf ears. In fact, the Communist Party of Yugoslavia (CPY) was in the midst of its own soul-searching, and its terms of debate mirrored the psychiatrists' to a very significant extent. In the immediate post-war years, the CPY grew increasingly concerned over the broader implications of the very revolutionary policies that it was implementing. Just as the Yugoslav psychiatric community doubted the ability of the partisans to assume high-ranking political and military positions, the CPY worried that its new inflated membership included large numbers of those unprepared or unable to fulfil its lofty revolutionary mission. Although the CPY never referred to ‘hysteria’ or ‘neurosis’ or commented directly on psychiatrists' pronouncements, it shared the concerns of the psychiatric community. Both recognised the same set of symptoms as undesirable and problematic: it was those comrades with underdeveloped political/ideological awareness and overdeveloped ambition and self-love that caused concern, which meant that that medical professionals as well as political commissars should be involved in the psychiatric treatment of such patients.^54^ The CPY criticised the same problematic tendency within its ranks, and its motives did not differ from the psychiatrists': it was deeply worried that the new revolutionary society promoted and rewarded those who did not deserve it. The ideological and the medical thus converged: the increased social mobility posed, ironically, a serious challenge to the core ideological premises of the Party. The partisan neurotic became much more than a medical or psychiatric category: he or she was merely the extreme version of one of the Party's most pervasive political and ideological problems of the immediate post-war period.

Before 1945, the Party experienced contradictory impulses: on the one hand, it needed to accept and convert as many people as possible regardless of their previous affiliations and ideological and moral credentials; on the other hand, throughout the war, the highest organs of the CPY constantly debated the most suitable ways to preserve the organisation's ideological purity and avoid any drastic fall in the membership's political awareness, commitment and capabilities. The wartime conditions in which the CPY underwent enormous growth and organised an ultimately very successful resistance struggle dictated the lowering of the entrance criteria for new members in the critical period of 1941–5. The Party boasted 141,066 members at the end of 1945; before it called for an uprising against the German forces in 1941, it had no more than 12,000 official followers.^55^ The membership, therefore, increased almost twelve times in a little over four years, and this was bound to provoke significant shifts within the Party's internal organisation, self-image and perception of its own mission. Even more importantly, the membership grew fastest in the military units, and the largest contingent of new Party card holders was made up of partisan soldiers. The Party admitted a number of peasants who quickly came to constitute the largest single social group within the organisation, and this provoked a wide-ranging internal debate regarding the precarious status of the CPY as a working-class vanguard. Moreover, the majority of new members and partisan fighters demonstrated a rather disappointing level of understanding of Marxism-Leninism, the CPY's goals, history of the workers' movements, etc. The widespread illiteracy among the masses of partisan soldiers constituted another colossal obstacle.^56^

The Party's vacillations grew even more complex in the aftermath of the war. The CPY could not straightforwardly criticise the trend of growing membership: in the final analysis, it would make it possible for revolutionary tasks to be completed more efficiently and quickly, and would involve as sizeable a part of the population as possible in building a new socialist society, which was in line with the Party's core tenets of democratic citizenship. In fact, the CPY functionaries often called for increasing its cadres ‘fifty-fold, a hundred-fold’, and warned against creating an isolated, elitist party organisation which would distrust the working-class masses and alienate them by placing itself above them. Growth of membership was essential if the Party was to preserve its authority, political influence, and take over the core institutions of the state. But the idea of a mass organisation remained distasteful to the CPY ideologues and leadership: in 1946, mass organisations were referred to as ‘institutions erected on clay foundations’, and the CPY did not want its sudden popularisation to result in ‘anarchy and laxity’.^57^ This was to put the Party in a rather difficult position: the idea of a large Communist Party, but made up exclusively of people with impeccable ideological prowess and political and cultural sophistication. Creating a mass cadre Party was to prove extremely difficult, and it would require a mammoth educational apparatus on the part of the CPY. In the meantime, the growing membership posed enormous problems for the Party's ideological commitment, and a far-reaching debate ensued which was meant to target the core difficulties in this regard, and profile the most problematic members.

Descriptions of the unsatisfactory performance of new members originated in war time and immediate post-war reports written by political commissars assigned to partisan units. These reports (analysed below) established the problematic character of a partisan fighter or Party member: usually a peasant, or a very low-skilled worker, exceptionally young, with low or no literacy, who only joined the Party after 1941 and had possibly previously been under the influence of some sort of anti-Marxist propaganda. He (or she) demonstrated an exceptionally low degree of political awareness and ideological (meaning Marxist) prowess, was frequently very ambitious and extremely sensitive to ‘comradely criticism’. He lacked discipline and self-control, and was generally a difficult person to cooperate with, either because of his disinterestedness and dullness, or the absence of respect for his comrades.

As Milovan Djilas, the Party's leading ideologue in this period, noted in 1946, ‘a number of new people entered the party, who brought with themselves a number of beliefs foreign to the party, a mass of illusions and prejudices.’^58^ The profile of the unsatisfactory partisan soldier as it emerged in internal Party reports throughout late 1943, 1944 and 1945 thus contained almost all of the major psychological characteristics of a typical partisan hysteric: ‘High degree of illiteracy, very little personal self-initiative, lack of responsibility for the unit as a whole, sick ambition for higher ranks and positions—these are the greatest flaws of [our] military commanders.’ These problems were then immediately related to the educational level and social background of the soldiers in question: ‘All these drawbacks were in most cases an inevitable phenomenon because our officers were mainly workers or peasants, almost without any education.’^59^ The core difficulties encountered in the partisan units were thus related to the extremely low level of political and ideological awareness, and to cultural and educational backwardness of the newly admitted: ‘the peasant element of [a mountain area in Bosnia] is shrewd and loyal to us, but insufficiently elevated politically, and … very backward in terms of the general culture and awareness.’^60^

The Communist Party officials were particularly concerned with what they commonly perceived as the exceptional ambitiousness of many recently enrolled members and soldiers: after 1945, they feared that many had joined not out of a true, selfless commitment to the Party's goals, but for egocentric and opportunistic reasons. The war neurotic was also characterised by his relentless desire for recognition and advancement. Partisan neurosis was by definition the condition of the overly ambitious, who considered their own social expectations to have been unjustly disappointed. In a report of the Party's Ideological Commission following a visit to several local party organisations in Vojvodina in 1947, the authors placed particular emphasis on the need to get rid of the ‘opportunistic elements’, who, as local leaders, ignored democratic forms of governance and frequently expressed arrogance and authoritarianism. Careerism and the tendency of certain sections of the Party membership to avoid more complex duties were the core problems, which showed some high-ranking members' disturbing lack of a true commitment to the Party's revolutionary aims.^61^

Certain traits, such as low self-criticism and difficulties with accepting negative critiques, automatically signified the ‘absence of the Communist values’. In personal references for several young Party officers towards the end of the war, a political commissar denounced one soldier's ‘absence of self-criticism and a low commitment to the Party; he overestimates himself and frequently underestimates others. There is a lot of egocentrism in him, bragging, often tells untrue stories.’^62^ Another partisan was described as ‘extremely sensitive, every irregularity towards him upsets him very much’.^63^ This heightened sensitivity to negative evaluations was defined as one of the core sources of neurotic conflicts in traumatised partisans: they reacted violently to any criticism, frequently insisted on their own values and achievements (often through demonstrative seizures), and were described by virtually all medical doctors involved in the discussion as egotistic and self-centred. Another Party member was ‘sensitive to a fair degree, so that even today he has not forgotten some insults by certain people at the beginning of the uprising, although some of these people are nowadays at responsible positions’.^64^

In response to these increasingly disconcerting ideological and pragmatic conundrums, both during and after the war, the Party's higher echelons insisted on devising and implementing a comprehensive programme of cultural enlightenment and raising the partisans' general educational level through Party courses, political and ideological lectures and discussion groups, and campaigns for fighting illiteracy. This was believed to be the ultimate strategy for a thorough cultural and political re-education of Party and partisan neophytes, and their gradual moulding into satisfactory Communists. In this sense, the educational approach corresponded to Klajn's recommendations; in the interpretation of the Central Committee and various political commissars working in partisan brigades and divisions, the problem resulted from a low general cultural level, denied access to any meaningful forms of political enlightenment, and protracted exposure to the ‘enemy propaganda’.

The CPY consequently engaged in implementing a mammoth educational operation. Even during the war, each partisan unit had its own commissioner in charge of ideological-political development of the soldiers, who organised historical and political lectures and courses. After the war this activity intensified and became more centralised. In 1946, the Central Committee of the CPY concluded that the core political problems of the post-war were ‘insufficient commitment to a pure ideological line within the Party’, and weak, unsystematic and uninspired educational, cultural and ideological work with the working class, peasants and intellectuals.^65^ Djilas instructed the Party organs to avoid formalistic approaches to the ideological ‘elevation of the masses’, and to devise differentiated political-educational programmes for working with different groups of the population. The Party's responses were to develop and sponsor evening political schools and distance-learning for all Party functionaries, regular seminars at every level and in every cell of the CPY organisation, kruzhoks to discuss and clarify the most important political events of the time, conferences and consultations, educational film screenings, and to pay particular attention to the educational role and potential of newspapers.

This massive educational and cultural work was to target non-members as well, and to be also implemented through non-Party mass organisations, such as the Communist Youth Association, women's organisations, trade unions and cultural organisations in the countryside (from where some of the most politically illiterate new members came). Following the early Bolshevik model of educational policy, the CPY set for itself a vast enlightenment mission, and strove to turn the entire country into a large cultural-educational institution. The powerful new apparatus of the CPY's Central Committee, Agit-Prop (agitation and propaganda), was in charge of authoring, controlling and centralising the country's cultural and educational politics, and co-ordinated the activities of educational officers in every Party cell. In 1945, for instance, those in charge of Agit-Prop in factories, countryside and schools were advised to set up reading rooms and chess clubs, instigate and oversee newspaper publishing, organise theatrical performances and other forms of cultural events, as well as seminars and materials for studying Marxism-Leninism.^66^

On the other hand, the much less inclusive approach characteristic of Dojč also had its proponents: Party organs occasionally insisted on the ideological strengthening and internal ‘cleansing’ of the organisation at all levels. Even those like Djilas, who committed themselves wholeheartedly to efforts at popular education, maintained that the Party's inclusiveness must have clear boundaries, and that ‘theoretical fight and ideological purity’ remained the most important values of the post-war period.^67^ After the liberation and revolutionary war were completed successfully, the Yugoslav Party and society were left with an equally important task: to weed out every person and every habit which could seriously endanger the full overhauling of the economic and political system. The Party's ideologues used every opportunity to emphasise that the post-war years were necessarily marked by a continuing struggle—against the social forces of ‘old Yugoslavia’, their liberal and capitalist preferences, their dangerous and anti-socialist intentions and qualities: corruption, black marketeering, speculation and exploitation. So the immediate post-war period was marked by frequent Party purges, especially in late 1945 and in 1946, which were meant to ‘relieve’ the CPY of ‘accidental and ideologically-politically immature’ new members. Moreover, these years also saw a flurry of the Party's internal and public communications, which warned about the growing problem of ‘petty-bourgeois’ and ‘bureaucratic’ tendencies within the organisation. These included careerism, egotism, unbridled ambition, selfish materialism, laziness, boastfulness and vainglory, dishonesty, sloppiness and irresponsibility, and the immoral struggle for political/economic positions.^68^ These trends were deeply upsetting to the CPY's ideologues, as they harmed the image of the Party as the beacon of new socialist morality. Individual members accused of such behaviour were regularly excluded from the CPY throughout the late 1940s, and this alternative campaign to preserve its moral integrity and political-ideological ‘purity’ was even more important for lowering the Party's membership than even conventional mass purges. The basic dilemma—whether to educate or eliminate the less than ideal new members—thus mirrored the psychiatric discussions on strategies for integrating the war neurotic into Yugoslavia's changing society.

## Conclusion

In 1957, a group of Zagreb-based psychiatrists, led by Betlheim, conducted a follow-up study of wartime neurotic patients, aiming to inquire into their adaptation to civilian life in the course of ten or so years after the end of the war. After interviewing 34 former patients, who had all received treatment in military hospitals after the end of the war, the psychiatrists concluded that in the majority of cases the former partisans had suffered from ‘superficial neurosis’ that did not harm deeper layers of their personality, and consequently they faced no larger problems reintegrating into the post-war society. This was true particularly for those interviewees who were younger than 18 at the time of their seizures: according to the study, they overcame their neurotic disorders very easily, since those appeared to be just a phase in the maturation and development of their personality.^69^ The authors recommended superficial psychotherapy, with particular attention to mental hygiene measures and prevention. In their conclusion, this group of eminent military psychiatrists argued that the outbreak of ‘partisan hysteria’ did not seem to have left any deeper wounds in Yugoslav society: the former neurotics apparently shed their neurotic condition fairly quickly and were able to adapt to the peacetime circumstances without major disturbances. They were cured: they outgrew their ‘hysteria’, leaving it behind in the course of their personal development, education and perhaps also upward social ascent.

In the long term, the therapeutic engagement with ‘partisan hysteria’ permanently transformed military psychiatry. After the end of the war, Dojč had worried that indulging ‘partisan neurotics’ would result in the fixation of their neurosis. However, a decade or so later there was a growing belief among Yugoslavia's military psychiatrists that partisan war trauma indeed left no serious or long-term consequences, that the disorder could be dealt with efficiently and terminally, and the patients could emerge fully rehabilitated. But this latter assumption was based on concepts radically different from those initially proposed by Dojč and his associates. Military psychiatry was now not there simply to diagnose defects. It was also supposed to directly address and eliminate them without writing anybody off.^70^

As the follow-up discussions regarding partisan neurosis demonstrated, the fears of traditional military psychiatrists never materialised: the partisan neurotics did not prove to be constitutionally inferior, or psychopathic personalities disruptive of the social order; on the contrary, their gradual full reintegration in a developing socialist society struck Betlheim and his colleagues as quite miraculous. At the same time, psychoanalysts used the disorder quite successfully to bolster the importance of their own field, and, in the course of the 1940s and 1950s, they emerged as the most important participants in this military psychiatric debate. By the early 1950s, psychotherapy based on dynamic principles was making significant inroads into Yugoslavia's psychiatry.^71^ In the military sphere as well, leading psychiatrists began to criticise purely biomedical approaches, and to recommend taking into consideration not only patients' constitutional predilections but also their socioeconomic background, life experiences as well as their internal psychological universes.^72^ In these psychiatrists' interpretations, psychotherapy and dynamic psychiatry were far more efficient in curing (or in any case, improving) patients than were the more conservative therapies based solely on medication and somatic treatment. Moreover, in a country as small as Yugoslavia, the military could not afford to discard large numbers of potential soldiers due to psychiatric difficulties, most notably the epidemic of partisan hysteria.^73^ By the late 1950s, in fact, psychiatrists spoke of the Yugoslav army as an institution ideally suited to socialise and re-educate young men with psychological or emotional problems. For these reasons, military psychiatry needed to step in and grow stronger. Its aims, though, would now be different from Dojč's early urges to screen and eliminate those prone to neurosis.^74^ This new military psychiatry was to cure.

## Funding

Funding to pay the Open Access publication charges for this article was provided by Wellcome Trust.

## Footnotes

1 Arhiv Sanitetske sluzbe Ministarstva odbrane, R-19, ‘Neuropatija (slicna histeriji)’, 1–2; unless otherwise stated, all translations from Serbo-Croatian and German are mine.

2 Hugo Klajn, *Ratna neuroza Jugoslovena* (Belgrade: Tersit, 1995—reprint, originally published in 1955), 65–6.

3 Military psychiatrists, who often doubted the reality of the disorder, contested the thesis that the partisan hysterics experienced an altered state of consciousness during their seizures, and argued instead that the entire ‘performance’ was fully conscious and pre-planned.

4 See, for instance, Ben Shephard, ‘Pitiless Psychology: The Role of Prevention in British Military Psychiatry in the Second World War’, *History of Psychiatry*, 1999, 10, 491–524; T. Copp and B. McAndrew, *Battle Exhaustion: Soldiers and Psychiatrists in the Canadian Army 1939–1945* (Montreal: McGill/Queen's University Press, 1990); Edgar Jones and Stephen Ironside, ‘Battle Exhaustion: The Dilemma of Psychiatric Casualties in Normandy, June-August 1944’, *Historical Journal*, 2010, 53, 109–28; N. Thalassis, ‘Soldiers in Psychiatric Therapy: The Case of Northfield Military Hospital 1942–46’, *Social History of Medicine*, 2007, 20, 351–68; Hans Pols, ‘The Tunisian Campaign, War Neuroses, and the Reorientation of American Psychiatry during WWII,’ *Harvard Review of Psychiatry*, 2011, 19, 313–20.

5 Michal Shapira, ‘The Psychological Study of Anxiety in the era of the Second World War’, *Twentieth Century British History*, 2013, 24, 31–57; Ruth Leys, *Trauma—A Genealogy* (Chicago: University of Chicago Press, 2000), 191.

6 Paul Lerner, *Hysterical Men: War, Psychiatry and the Politics of Trauma in Germany, 1890–1930* (Ithaca: Cornell University Press, 2003).

7 Julie V. Brown, ‘Revolution and Psychosis: The Mixing of Science and Politics in Russian Psychiatric Medicine, 1905–1913’, *Russian Review*, 1986, 46, 283–302; on popular and psychiatric conceptualisations of war trauma in Russia, see also Catherine Merridale, ‘The Collective Mind: Trauma and Shell-Shock in Twentieth-Century Russia’, *Journal of Contemporary History*, 2000, 35, 39–55.

8 Klajn, *Ratna neuroza Jugoslovena*, 68.

9 *Ibid*., 100.

10 *Ibid*., 78.

11 *Ibid.*, 66–8.

12 Stanislav Piscevic, *Sanitetska služba u narodno-oslobodilačkom ratu Jugoslavije 1941–1945 II* (Belgrade: Vojnoizdavački i novinski centar, 1989), 108–11.

13 Isak Alfandari, ‘Ratna neuroza’, *Vojno-Sanitetski Pregled (VSP)*, 1944, 4–5, 119–22.

14 Josip Dojč, ‘Inozemstvo-Mania, nova zarazna bolest!’, Arhiv Sanitetske sluzbe Ministarstva odbrane, R-202.

15 Arhiv Sanitetske sluzbe Ministarstva odbrane, R-19, ‘Podaci o ratnim neurozama’, 7 December 1944, 246.

16 In this article, by ‘psychiatrists’ I refer to both organicists (adhering to the biomedical, somatic models of psychiatry and mental illness) and psychoanalytically, psychodynamically or psychotherapeutically oriented psychiatrists. In Yugoslavia (and the rest of the region), the separation line between psychoanalysis and psychiatry was often blurred, as virtually all psychoanalysts were originally trained as psychiatrists and usually maintained both practices. Psychoanalytically trained practitioners, as well as those adhering to psychodynamic and psychotherapeutic principles and psychogenic interpretations of the origins of mental pathology, constituted a more or less discrete subgroup within the psychiatric discipline, while military psychiatry was dominated by organicist theories in the early post-war period and well into the 1950s. Importantly, Yugoslavia's interwar psychiatry was almost exclusively biomedical, with exceptional figures such as Klajn and Betlheim fairly marginalised.

17 Stjepan Betlheim, ‘O ratnim neurozama’, *Glas Udarnika: List V korpusa Jugoslavenske Armije*, 1945, 3, 92.

18 Petar Klajn, *Razvoj psihoanalize u Srbiji* (Belgrade: Pedagoska akademija, 1989), 66.

19 Although the Institute itself was rather short-lived mainly due to logistical problems, the psychoanalytic approach to the problem of partisan hysteria persevered in most other psychiatric institutions in Yugoslavia. Even though they were initially attacked by military psychiatrists, Klajn's and many of his followers' publications and experiments from the early post-war period remained very influential and ultimately transformed the psychiatric understanding and therapy of war trauma: in the long run, it was the dynamic, psychotherapeutic approach that proved more effective in treating the disorder, and in the 1950s it was considered to hold more explanatory value than organicist theories for a number of reasons. See V. Kovacevic, ‘Pregled istorijskog razvoja dinamicke psihijatrije u Srbiji’, *Avalske sveske*, 1983, 6, 179–89. In the end, as I will discuss in the final section of the article, the leading military psychiatric institutions in the country adopted the psychodynamic approach as the foundation of their medical military doctrine.

20 See S. Milanovic *et al.*, ‘Historical Development of Psychiatry in Serbia’, *Psychiatria Danubina*, 2009, 21, 156–65.

21 Dezider Julius, ‘Nova nastojanja u lijecenju neuroza’, *Lijecnicki Vjesnik*, 1951, 5, 90.

22 P. Klajn*, Razvoj psihoanalize u Srbiji*, 66. Klajn published his book with the Headquarters of the Medical Service of the Yugoslav Military in 1955, but military censorship held the publication in check for a rather long period of time: the manuscript had been submitted as early as 1945.

23 Alfandari, ‘Ratna neuroza’, 119–22; while the war was still being fought, Alfandari published the first article on the problem of the partisans' war neurosis in a medical journal. He invested significant efforts in proving that the kind of disorder encountered among Yugoslav resistance fighters existed in other European armies in the past, and was even a well-known psychiatric condition of nineteenth-century soldiers, theorised by Charcot. It was opinions such as Šalek's that Alfandari was contesting: Šalek's ideologically slanted explanation argued that this form of war trauma could only have appeared in an egalitarian army and socialist community; in fact, ‘partisan hysteria’ proved that the partisans knew they were fighting for their own interests rather than those of an estranged state and its ruling class—because of this their war trauma did not manifest itself as tremor or anxiety states, and the number of traumatised soldiers in the Second World War was significantly smaller than in the First World War (Arhiv Sanitetske sluzbe Ministarstva odbrane, R-19, ‘Podaci o ratnim neurozama’, 247).

24 Zarko Trebjesanin, ‘Klajnova analiza ratne neuroze’, in Hugo Klajn, *Ratna neuroza Jugoslovena*, 12–30.

25 Klajn, *Ratna neuroza Jugoslovena*, 39.

26 Stjepan Betlheim, ‘O ratnim neurozama’, *Glas Udarnika: List V korpusa Jugoslavenske Armije*, 1945, 3, 91.

27 Klajn, *Ratna neuroza Jugoslovena*, 111–12.

28 *Ibid*., 95.

29 *Ibid*., 48.

30 *Ibid*., 84.

31 *Ibid*., 88–9.

32 *Ibid*., 42.

33 *Ibid*., 17–18.

34 *Ibid*., 81–2.

35 *Ibid*., 78.

36 *Ibid*., 82.

37 *Ibid*., 149.

38 Under the circumstances, Betlheim experienced a quick change of heart, and his initially sympathetic attitude towards partisan neurotics was to transform into an approach rather close to Dojč's therapeutic pessimism. In October 1945, Betlheim wrote to his wife from Kovin: ‘I keep hoping that the hospital would be disbanded soon. It is impossible to work here. The personnel is dispersing, nobody dares to do anything against them [the patients]. They break windows, cause incidents in the town, terrorise everybody. We already have 130 of them here. What would it be like if there were 500! Impossible to imagine.’ Quoted in: Ruth Betlheim and Gordana Lerotic, *Stjepan Betlheim: Radovi, pisma, dokumenti, 1898–1970* (Zagreb: Antibarbarus, 2006), 12.

39 Klajn, *Ratna Neuroza Jugoslovena*, 151.

40 *Ibid*., 149.

41 Josip Dojč, ‘O biti zivcanih napadaja u ratu (ratna neuroza)’, *Vojno-Sanitetski Pregled (VSP),* 1946, 3, 118.

42 Stjepan Betlheim, ‘Samoostecenja kod ratnih neuroticara’, *VSP*, 1946, 3, 331–3.

43 Ruth Betlheim and Gordana Lerotic, *Stjepan Betlheim: Radovi, pisma, dokumenti, 1898–1970* (Zagreb: Antibarbarus, 2006), 100. In the years following the failed experiment in Kovin, Betlheim returned to Zagreb and worked closely with physicians of the psychiatric ward of Zagreb's military hospital, where his much harsher and more pessimistic attitudes towards re-educating and recovering partisan neurotics were certainly affected by his disappointment in Klajn's pedagogical methods in Kovin (see footnote 38). However, by the early 1950s, Betlheim had re-established himself as the leading psychoanalyst and psychotherapist in socialist Yugoslavia, having become the head of the psychiatric ward of the Zagreb University Neuro-Psychiatric clinic, joined the International Psychoanalytic Association, founded the first out-patient psychotherapeutic ward in Croatia at the Zagreb Faculty of Medicine, and become a leading scholar and practitioner of psychoanalytically informed group therapy for sexual disorders in Yugoslavia, and his views on the issue of partisan neurosis evolved yet again.

44 *Ibid*., 332.

45 See, for instance, Neuro-psychiatric hospital Vrapce, Archive, file 4390/45, Bogdan Gacpar, diagnosed with ‘obtuseness’ bordering on ‘feeble-mindedness’; file 4249/45, Zdravko Gajic; file 4504/45, Slavko Boromisa, who was described as ‘primitive’, incapable of understanding abstract notions, with limited understanding and interpretation skills, unable to read and count properly, unable to construct coherent sentences; file Milos Koncar, suspected of schizophrenia as well as psychopathia gravis, also had ‘characteristically empty facial expressions’, ‘scant knowledge and weak intelligence’ which he ‘attempted to conceal with superficial scientific phrases and political slogans’; file 4060/45, Milan Grguric, whose mother was reported to have suffered ‘from nerves’ as well.

46 Betlheim and Lerotic, *Stjepan Betlheim*, 95–7.

47 Josip Dojč, ‘Simulacija i simulanti sa psihijatrijskog gledista’, *Srpski arhiv za celokupno lekarstvo*, 1947, 12.

48 Dojč, ‘O biti zivcanih napadaja u ratu’, 119.

49 *Ibid*., 118.

50 ‘Samoostecenja kod ratnih neuroticara: Diskusija,’ *VSP*, 1946, 7–8, 334.

51 Josip Dojč*,'*Intelektualni i psihicki defekti u armiji', *VSP,* 1952, 9, 300.

52 *Ibid*.

53 Josip Dojč, ‘Ratne psihoneuroze sa gledista savremene vojne psihijatrije’, *VSP*, 1952, 7–8, 233.

54 Arhiv Sanitetske sluzbe Ministarstva odbrane, R-19, Dr Šalek, ‘Podaci o ratnim neurozama’, 243.

55 Branko Petranovic, *Istorija Jugoslavije, knjiga III: Socialisticka Jugoslavija 1955–1988* (Belgrade: Nolit, 1980), 39.

56 On the the relationship between peasants and the Communist Party of Yugoslavia during and after the war, see Melissa Bokovoy, *Peasants and Communists: Politics and Ideology in the Yugoslav Countryside, 1941–1943* (Pittsburgh: Pittsburgh University Press, 1998); Milovan Djilas, *Wartime* (Mariner Books, 1980); Fikreta Jelic-Butic, *Hrvatska seljacka stranka* (Zagreb: Globus, 1983).

57 Petranovic, *Istorija Jugoslavije*, 39–41.

58 Milovan Djilas*, O danasnjim zadacima partije* (Zagreb: Naprijed, 1946), 20.

59 Archive of Yugoslavia, *Izvori za istoriju SKJ*, book 18, 243.

60 *Ibid*., 306.

61 Archive of Yugoslavia, KPJ, fund 507, Ideoloska Komisija II/6-(1–71), 5–6.

62 *Izvori*, book 18, doc. 45.

63 *Izvori*, book 18, 318.

64 Archive of Yugoslavia, F-107, CK KPJ, doc. 1944/660.

65 Branko Petranovic and Momcilo Zecevic, *Jugoslavija 1918–1988: Tematska zbirka dokumenata* (Belgrade: Izdavacka radna organizacija Rad, 1988), 878–86.

66 Petranovic, *Istorija Jugoslavije*, 120–39.

67 *Ibid*., 23.

68 *Ibid*., 40–1.

69 Stjepan Betlheim *et al.*, ‘Adaptacija ratnih neurotika’, *VSP*, 1957, 9, 508–9.

70 *Ibid*., 508–9.

71 Branko Kandic, ‘Problemi i stanje psihijatrijske sluzbe u savremenoj armiji’, *VSP*, 1956, 7–8.

72 *Ibid*., 376.

73 Mira Vrabic, ‘Znacaj psihickih poremecaja u savremenom ratu, njihova ucestalost i profil sa istoriskim osvrtom’, *VSP*, 1958, 3, 201.

74 For a more thorough discussion of post-war military psychiatry in Yugoslavia, see Vladislav Klajn, ‘Problem rehabilitacije—rehabilitacija neuroticara’, *VSP*, 1958, 7–8.

